# Near-Isogenic Barley Lines Show Enhanced Susceptibility to Powdery Mildew Infection Following High-Temperature Stress

**DOI:** 10.3390/plants11070903

**Published:** 2022-03-28

**Authors:** Judit Kolozsváriné Nagy, Ildikó Schwarczinger, Lóránt Király, Renáta Bacsó, Attila L. Ádám, András Künstler

**Affiliations:** Centre for Agricultural Research, Plant Protection Institute, ELKH, 15 Herman Ottó Str., 1022 Budapest, Hungary; nagy.judit@atk.hu (J.K.N.); schwarczinger.ildiko@atk.hu (I.S.); bacso.renata@atk.hu (R.B.); adam.attila@atk.hu (A.L.Á.); kunstler.andras@atk.hu (A.K.)

**Keywords:** barley, barley powdery mildew, combined stresses, disease resistance, heat stress, Mla12, Mlg, mlo5, Mlo, near-isogenic lines

## Abstract

Barley cultivation is adversely affected by high-temperature stress, which may modulate plant defense responses to pathogens such as barley powdery mildew (*Blumeria graminis* f. sp. *hordei,* Bgh). Earlier research focused mainly on the influence of short-term heat stress (heat shock) of barley on Bgh infection. In this study, our aim was to investigate the effects of both short- and long-term heat stress (35 °C from 30 s to 5 days) on Bgh infection in the barley cultivar Ingrid and its near-isogenic lines containing different powdery mildew resistance genes (*Mla12*, *Mlg*, and *mlo5*) by analyzing symptom severity and Bgh biomass with RT-qPCR. The expression of selected barley defense genes (*BAX inhibitor-1*, *Pathogenesis- related protein-1b*, *Respiratory burst oxidase homologue F2,* and *Heat shock protein 90-1*) was also monitored in plants previously exposed to heat stress followed by inoculation with Bgh. We demonstrated that pre-exposure to short- and long-term heat stress negatively affects the resistance of all resistant lines manifested by the appearance of powdery mildew symptoms and increased Bgh biomass. Furthermore, prolonged heat stress (48 and 120 h) enhanced both Bgh symptoms and biomass in susceptible wild-type Ingrid. Heat stress suppressed and delayed early defense gene activation in resistant lines, which is a possible reason why resistant barley became partially susceptible to Bgh.

## 1. Introduction

Plants in nature must simultaneously face several different stresses as abiotic and biotic stressors act in concert and both have a significant impact on plant health [1,2]. In fact, the interplay of abiotic and biotic factors may have a key influence on plant–pathogen interactions, in particular on the outcome of a given infection [3,4]. Heat is one of the most severe abiotic environmental stress factors that limit crop growth and productivity [5,6]. Barley (*Hordeum vulgare* L.) is the fourth most relevant cereal crop worldwide [7], and environmental stresses such as heat stress have a profound impact on barley production [8]. Importantly, numerous studies have shown that heat stress usually negatively affects key plant disease-resistance mechanisms [1]. Powdery mildew diseases are caused by a wide variety of obligate biotrophic fungal pathogens, generally with limited host ranges. Barley powdery mildew (*Blumeria graminis* f. sp. *hordei*; Bgh) is one of the very few of these pathogens capable of infecting a monocot grass species, barley, and causing serious economical yield losses worldwide [9,10]. Barley plants employ several types of preformed and inducible defenses to prevent, or at least limit, infections when attacked by Bgh. An effective defense of barley plants to Bgh is mediated by homozygous recessive alleles of the *Mildew locus o* (*Mlo*) [11]. This resistance is non-race specific and is effective against almost all powdery mildew isolates [12,13]. The resistance reaction is tightly linked to rapid cell wall remodeling and accumulation of the reactive oxygen species (ROS) hydrogen peroxide (H_2_O_2_) in epidermal cells in response to attempted fungal penetration [12,14,15]. Barley *mlo* mutants exhibit pleiotropic phenotypes that can negatively affect harvest yields [16]. However, it has been demonstrated recently that wheat *Tamlo-R32* mutants with a 304-kilobase pair-targeted deletion in the *Mlo-B1* locus retain crop growth and yields while conferring robust powdery mildew resistance [17]. Furthermore, in barley, a number of race-specific, dominant, and semi-dominant R genes (e.g., *Mla* and *Mlg*) [18] may also confer resistance to Bgh and are associated with the activation of the hypersensitive response (HR), i.e., localized cell death at attempted infection sites [19]. The interaction between Bgh and different R genes containing resistant and susceptible (*Mlo*) barley genotypes has been described by Hückelhoven and his coworkers [20]. After the first 16 h of infection, the fungus penetrates epidermal barley cells in susceptible and resistant Mla12 plants. During compatible interactions, cell wall penetration is followed by haustorium and elongated secondary hyphae formation [21]. However, in the epidermal cells of resistant *Mla12* genotypes, a spreading HR of mesophyll cells directly below the attacked epidermal cell arrests fungal development. On the other hand, in the *mlo5*-mediated resistance response, pathogen penetration is inhibited by effective and rapid cell wall remodeling (i.e., papilla formation). Furthermore, in cells of *Mlg* genotypes, the papilla formation is associated with a single-cell HR [20]. Because the defense reactions mediated by these R genes (*mlo5*, *Mlg,* and *Mla12*) act differently during Bgh infection, it is conceivable that high-temperature stress may cause distinct effects based on the presence of a given R gene. 

The duration of high-temperature stresses is another fundamental factor of plant–pathogen interactions. Heat shock (a short-term exposure to high temperatures), e.g., submerging plants in 49 °C water for 20 seconds (s) one day before Bgh inoculation, resulted in the susceptibility of near-isogenic lines of barley (*H. vulgare* cv. Ingrid) containing different resistance genes (*mlo5*, *Mlg*, *Mla12*). The same heat shock further increased susceptibility to Bgh in genetically susceptible barley (cv. Ingrid *Mlo*) [22]. We have shown that a similar heat shock (49 °C for 45 s) can partially suppress the symptomless nonhost resistance of barley to wheat powdery mildew (*B. graminis* f. sp. *tritici*) [23]. On the other hand, in certain cases, a short-term exposure to elevated temperatures may increase the plant’s resistance to pathogens. In barley (cv. Golden Promise), a heat shock of 50 °C for 60 s induces resistance against Bgh [24,25]. However, more prolonged high temperatures generally appear to decrease disease resistance in barley. For example, an exposure to 36 °C for 30 minutes (min), 60 min, and 120 min prior to pathogen inoculation confers enhanced susceptibility to Bgh in both genetically resistant (containing different *mlo* alleles) and susceptible barley cultivars [26]. Interestingly, wheat powdery mildew (*B. graminis* f. sp. *tritici*) growth was inhibited following an exposure to high temperatures (26–30 °C) for several days, and no typical powdery mildew symptoms were observed [27]. However, in this case, the absence of powdery mildew symptoms is likely due to the inhibition of pathogen growth at constant high temperatures rather than the activation of plant defense responses. We found that prolonged heat stress pre-exposure (24, 48, and 120 h at 35 °C) confers enhanced susceptibility to Bgh in a genetically susceptible barley line (MvHV 118-17), while a resistant line (MvHV 07-17) retains its pathogen resistance [28]. A robust reactivation of heat-suppressed defense gene expression in the barley line MvHV07-17 is a likely reason for retaining Bgh resistance even after a prolonged pre-exposure to 35 °C [28]. 

In plant cells, high temperatures may often interfere with several interconnected signaling pathways that cause, e.g., an initially enhanced ROS production contributing both to antioxidant induction (controlling ROS) and elevated expression of heat shock protein (HSP) genes that encode molecular chaperones stabilizing/protecting, e.g., antioxidant enzymes and immune receptors. Furthermore, heat-induced extracellular Ca^2+^ influxes through plasma membranes may activate heat shock transcription factors (HSFs) that regulate HSP expression. Ca^2+^ is also responsible for the activation of membrane-bound NADPH oxidases that produce ROS and contribute to the development of plant disease resistance. Importantly, the development of heat tolerance has been associated with the proper functioning of HSPs and antioxidant enzymes in several plant species (see [1,29] and references within). Although several responses can be identified that are common between heat stress and combined (heat and pathogen infection) stresses, generally, plant transcriptional responses induced by a combination of heat stress and pathogen infection are specific and unpredictable from responses to heat-stress application alone [1]. 

Recently, we found that prolonged heat stress pre-exposure confers enhanced susceptibility to Bgh in a genetically susceptible barley line, while pathogen resistance is retained in a resistant line [28]. Therefore, we hypothesized that prolonged heat stress may similarly increase the Bgh susceptibility of wild-type Ingrid (*Mlo* genotype), a genetically well-characterized barley cultivar. Furthermore, we assumed that cv. Ingrid near-isogenic lines containing different R genes may react differentially to high-temperature stress. Therefore, the objective of this study was to investigate the influence of short- and long-term heat stress (35 °C from 30 s to five days) on resistant and susceptible near-isogenic lines (Mlo, mlo5, Mla12, Mlg) of barley cv. Ingrid infected with Bgh race A6.

## 2. Results

### 2.1. Determination of the Effect of Heat Stress on Powdery Mildew Infection in Near-Isogenic Barley Lines

**Figure 1:**
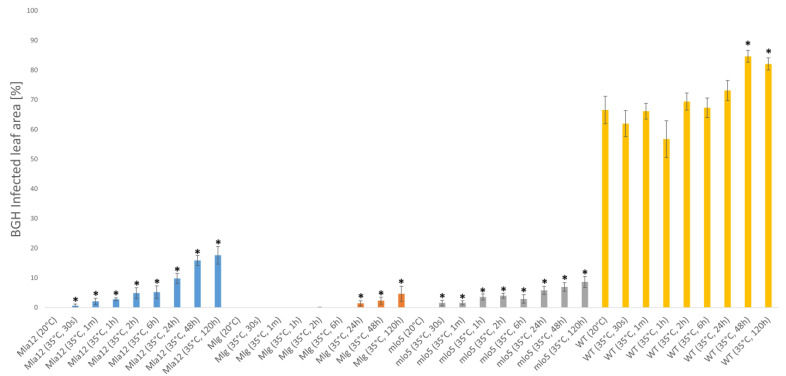
Evaluation of powdery mildew (*Blumeria graminis* f. sp. *hordei* A6, Bgh) symptoms in resistant (Mla12, Mlg, mlo5) and susceptible (WT) near-isogenic lines of *Hordeum vulgare* cv. Ingrid seven days after inoculation. Barley plants were exposed to high-temperature stresses (35 °C) for different periods of time (30 s to 120 h) before powdery mildew inoculation. Control plants were kept at 20 °C. Symptoms were calculated as the percentage of area covered by powdery mildew symptoms per leaf. The graphs show the average of three experiments. Error bars represent standard deviation. Asterisks (*) indicate statistically significant differences between non-heat-treated and heat-treated plants within the respective barley genotypes at *p* < 0.05.

Resistant (Mla12, Mlg, mlo5) and susceptible (wildtype, Mlo) near-isogenic barley lines of cv. Ingrid were used in our experiments to determine how heat stress influences the defense responses of barley to powdery mildew (*Blumeria graminis* f. sp. *hordei* A6, Bgh). Barley plants were exposed to a high temperature (35 °C) for different durations (30 s, 1 min, 1 hour [h], 2 h, 6 h, 24 h, 48 h, 120 h) immediately before inoculation with Bgh. Disease symptoms were visually evaluated at seven days after powdery mildew inoculation. Our results showed that a significant increase in the proportion of leaf area covered by powdery mildew was detectable in plants previously exposed to 35 °C, as compared to plants maintained at 20 °C (Figure 1).

**Figure 2:**
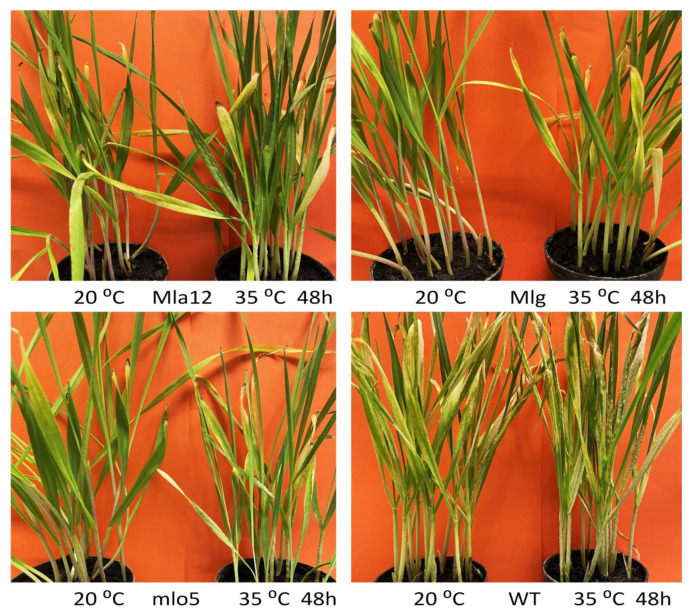
Powdery mildew (*Blumeria graminis* f. sp. *hordei* A6) symptoms in resistant (Mla12, Mlg, mlo5) and susceptible (WT) near-isogenic lines of *Hordeum vulgare* cv. Ingrid seven days after inoculation. Barley plants were exposed to high-temperature stresses at 35 °C for 48 h (h) before powdery mildew inoculation. Control plants were kept at 20 °C.

In the susceptible (WT) and one of the resistant lines (Mlg), only long-term (24, 48, and 120 h) heat stress could increase powdery mildew coverage, and no significant enhancement of powdery mildew symptoms was observed following heat exposure for less than 24 h at 35 °C (Figure 1). In contrast, in the resistant lines Mla12 and mlo5, shorter durations of heat treatment (from 30 s to 6 h) increased the powdery mildew coverage significantly. Moreover, along with the increase in the duration of heat stress, powdery mildew coverage also increased in both resistant and susceptible lines. In the barley lines Mla12, mlo5, and WT, exposure to high temperatures (35 °C) resulted in the appearance of powdery mildew symptoms not only on the leaf but also on the stem (Figure 2).

**Figure 3:**
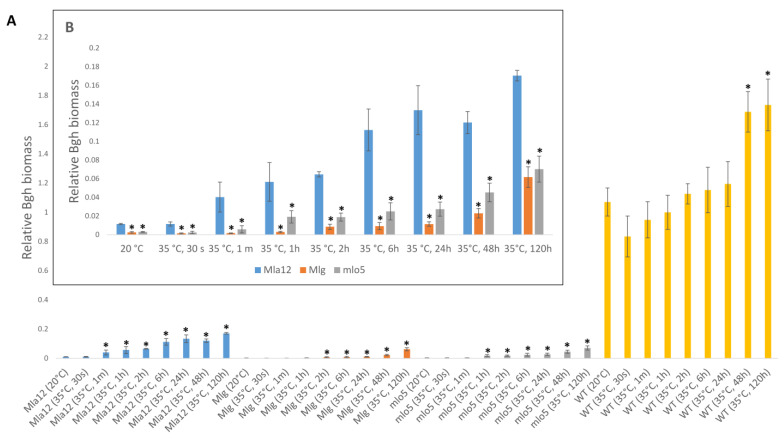
Relative powdery mildew (*Blumeria graminis* f. sp. *hordei* A6, Bgh) biomass (**A**) in resistant (Mla12, Mlg, mlo5) and susceptible (WT) lines of *Hordeum vulgare* cv. Ingrid seven days after inoculation. Barley plants were exposed to high-temperature stresses (35 °C) for different periods of time (30 s to 120 h) before powdery mildew inoculation. Control plants were kept at 20 °C. The graphs show the average of three experiments. Error bars represent standard deviation. Asterisks (*) indicate statistically significant differences between non-heat-treated and heat-treated plants within the respective barley genotypes at *p* < 0.05. (**B**) Relative Bgh biomass in resistant lines (Mla12, Mlg, mlo5) of *H. vulgare* cv. Ingrid seven days after inoculation. Asterisks (*) indicate statistically significant differences between Mla 12 and the two other resistant lines (Mlg, mlo5) within the respective treatment at *p* < 0.05.

In Mla12 barley, optimal temperatures (20 °C) activate a defense mechanism, the hypersensitive response (HR), which is characterized by a rapid cell death (local necrotic symptoms) at the point of pathogen ingress (Figure 2). However, in heat-stressed plants, HR symptoms are suppressed and fewer and smaller necrotic spots are observed, parallel to the appearance of powdery mildew symptoms (Figure 2). Overall, the proportion of powdery mildew-covered areas significantly increased at high temperatures in all investigated lines. The largest increase in powdery mildew coverage was observed in the Mla12 line while the Mlg line displayed the smallest effect in response to heat stress. In addition to symptom assessment, the quantification of Bgh was also performed by RT-qPCR. Results of the RT-qPCR assay supported the findings of the symptom assessment. Our results showed that significant increases in Bgh biomass are detectable in all investigated lines previously exposed to 35 °C, as compared to plants kept at 20 °C (Figure 3). 

Importantly, a significant increase in Bgh biomass was detectable in the susceptible line (WT) previously exposed to 35 °C for at least 48 and 120 h. In contrast, even shorter durations (from 1 min in Mla12, 1 h in mlo5, and 2 h in Mlg lines) of heat stress successfully increased powdery mildew biomass in resistant lines (Figure 3A). Remarkably, in Mla12, Bgh biomass was substantially higher than in the other two resistant lines (Mlg, mlo5) following each specific treatment (Figure 3B). In fact, the amount of fungal biomass in Mla12 barley is significantly higher even at 20 °C (without heat stress) as compared to the other two resistant lines (Figure 3B). The partial (incomplete) resistance provided by the *Mla12* gene and manifested as a spreading mesophyll HR, may explain this phenomenon [20].

### 2.2. Expression of Barley Defense/Stress Genes in Response to Heat Stress and/or Bgh Infection

**Figure 4:**
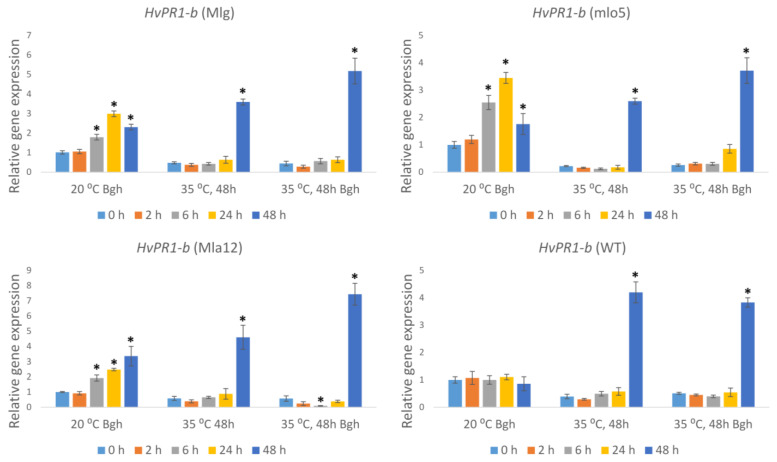
Expression of the barley *Pathogenesis related-1b* (*HvPR1-b*) gene as detected by RT-qPCR in resistant (Mlg, mlo5, Mla12) and susceptible (WT) *Hordeum vulgare* cv. Ingrid near-isogenic lines at early time points (0 h, 2 h, 6 h, 24 h, and 48 h) following powdery mildew (*Blumeria graminis* f. sp. *hordei* A6, Bgh) inoculation and/or heat stress. Heat stress treatments at 35 °C for 48 h were applied immediately before Bgh inoculation. Inoculated but not heat-treated (20 °C Bgh) and heat-treated but not inoculated (35 °C for 48 h) plants were used as controls. The graphs show the average of three experiments. Error bars represent standard deviation. Asterisks (*) indicate statistically significant differences from 0 h for each specific treatment at *p* < 0.05.

In order to assess the combined effects of heat stress and powdery mildew infection on activities of well-characterized barley stress/defense-associated genes related to Bgh infection such as *Pathogenesis related 1-b* (*HvPR1-b*), *Heat shock protein 90-1* (*HvHsp90-1*), *BAX inhibitor-1* (*HvBI-1*), and *Respiratory burst oxidase homologue F2* (*HvRBOHF2*), we assayed their expression in both resistant and susceptible plants inoculated with Bgh and previously exposed to 35 °C for 48 h. As controls, the defense gene expression was also assayed in plants that received only heat treatment but no Bgh inoculation and inoculated plants that were held at an optimal temperature (20 °C). Defense gene expression was detected following 48 h of heat stress treatment because this duration of heat stress has a significant impact on increasing Bgh symptoms and biomass in all investigated lines (see Figure 1 and Figure 3). In the Bgh-inoculated susceptible line (WT) held at 20 °C, the expression of *Pathogenesis related-1b* (*HvPR1-b*) did not change significantly in response to Bgh infection (Figure 4). In contrast, the resistant lines showed an enhanced *HvPR1-b* expression at 6, 24, and 48 h after inoculation, as compared to 0 h controls (Figure 4).

Heat stress and heat stress followed by Bgh inoculation significantly reduced the expression of *HvPR1-b* in both susceptible and resistant lines at early time points (from 0 to 24 h). However, 48 h after heat stress, the expression of *HvPR1-b* increased in all investigated lines. In fact, heat stress followed by Bgh infection further increased the expression of *HvPR1-b* at 48 h but only in resistant lines (Figure 4). 

**Figure 5:**
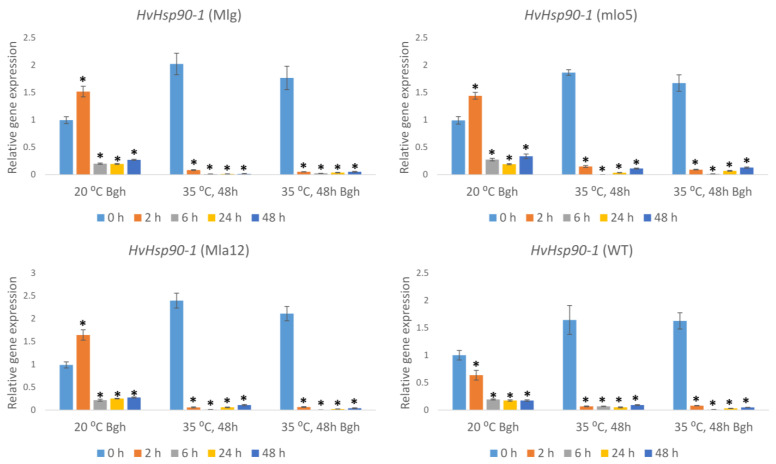
Expression of the barley *Heat shock protein 90-1* (*HvHsp90-1*) gene as detected by RT-qPCR in resistant (Mlg, mlo5, Mla12) and susceptible (WT) *Hordeum vulgare* cv. Ingrid near-isogenic lines at early time points (0 h, 2 h, 6 h, 24 h, and 48 h) following powdery mildew (*Blumeria graminis* f. sp. *hordei* A6, Bgh) inoculation and/or heat stress. Heat stress treatments at 35 °C for 48 h were applied immediately before Bgh inoculation. Inoculated but not heat-treated (20 °C Bgh) and heat-treated but not inoculated (35 °C for 48 h) plants were used as controls. The graphs show the average of three experiments. Error bars represent standard deviation. Asterisks (*) indicate statistically significant differences from 0 h for each specific treatment at *p* < 0.05.

In the Bgh-inoculated susceptible WT line maintained at 20 °C, the expression of *HvHsp90-1* was suppressed during the first 48 h of infection. On the other hand, in the resistant lines (Mla12, Mlg, and mlo5), the expression of *HvHsp90-1* was transiently induced 2 h after Bgh inoculation as compared to the zero-hour plants. After 2 h of early induction, *HvHsp90-1* expression was significantly suppressed by Bgh infection at later time points (Figure 5). 

Heat stress and heat stress followed by Bgh inoculation doubled the expression of *HvHsp90-1* in both resistant and susceptible lines at 0-h time points (after 48 h of heat stress) compared to plants held at 20 °C. The expression of *HvHsp90-1* was drastically suppressed at later time points (2, 4, 6, 24, and 48 h) in all investigated lines (Figure 5). Presumably, heat stress amplified the suppression of *HvHsp90-1* caused by Bgh infection.

**Figure 6:**
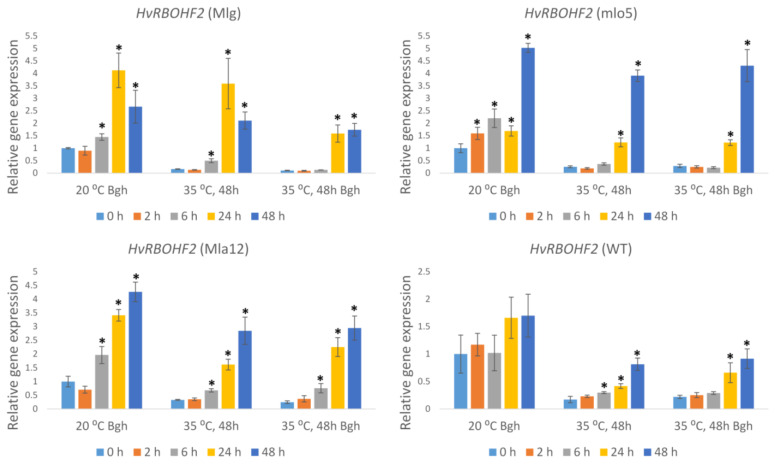
Expression of the barley *Respiratory burst oxidase homologue F2* (*HvRBOHF2*) gene as detected by RT-qPCR in resistant (Mlg, mlo5, Mla12) and susceptible (WT) *Hordeum vulgare* cv. Ingrid near-isogenic lines at early time points (0 h, 2 h, 6 h, 24 h, and 48 h) following powdery mildew (*Blumeria graminis* f. sp. *hordei* A6, Bgh) inoculation, and/or heat stress. Heat stress treatments at 35 °C for 48 h were applied immediately before Bgh inoculation. Inoculated but not heat-treated (20 °C Bgh) and heat-treated but not inoculated (35 °C for 48 h) plants were used as controls. The graphs show the average of three experiments. Error bars represent standard deviation. Asterisks (*) indicate statistically significant differences from 0 h for each specific treatment at *p* < 0.05.

*Respiratory burst oxidase homologue* genes encoding NADPH oxidase enzymes are a major source of the reactive oxygen species superoxide (O_2_^•−^) in plants. In the Bgh-inoculated susceptible line (WT) held at 20 °C, the expression of *HvRBOHF2* did not change significantly in response to Bgh infection (Figure 6). Conversely, the resistant lines showed an early and enhanced *HvRBOHF2* expression at 2, 6, 24, and 48 h in the mlo5 line and at 6, 24, and 48 h after inoculation in Mlg and Mla12 plants (Figure 6). 

Heat stress and heat stress followed by Bgh inoculation significantly suppressed and/or delayed the expression of *HvRBOHF2* in all investigated lines, compared to plants held at 20 °C (Figure 6). 

**Figure 7:**
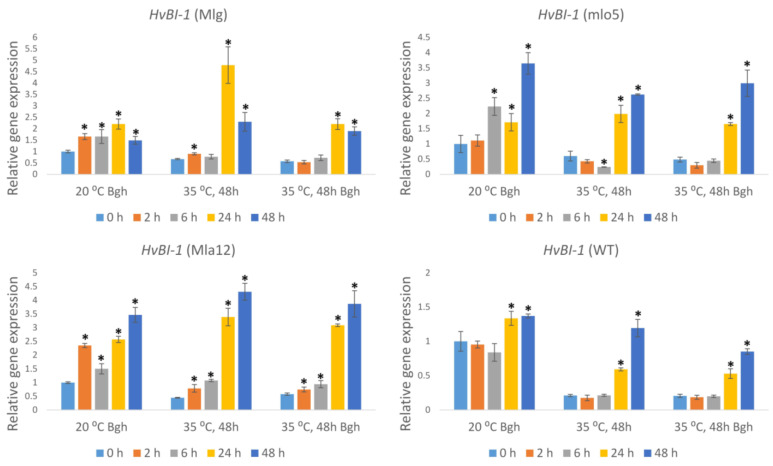
Expression of the barley *Bax inhibitor-1* (*HvBI-1*) gene as detected by RT-qPCR in resistant (Mlg, mlo5, Mla12) and susceptible (WT) *Hordeum vulgare* cv. Ingrid near-isogenic lines at early time points (0 h, 2 h, 6 h, 24 h, and 48 h) following powdery mildew (*Blumeria graminis* f. sp. *hordei* A6, Bgh) inoculation and/or heat stress. Heat stress treatments at 35 °C for 48 h were applied immediately before Bgh inoculation. Inoculated but not heat-treated (20 °C Bgh) and heat-treated but not inoculated (35 °C for 48 h) plants were used as controls. The graphs show the average of three experiments. Error bars represent standard deviation. Asterisks (*) indicate statistically significant differences from 0 h for each specific treatment at *p* < 0.05.

The expression of the cell death regulator *BAX inhibitor-1* (*HvBI-1*) showed a similar trend to that observed for *HvRBOHF2.* However, in the Bgh-inoculated susceptible line (WT) held at 20 °C, the expression of *HvBI-1* increased slightly but significantly at later time points (24 and 48 h) (Figure 7). Conversely, the resistant lines showed an early and enhanced *HvBI-1* expression starting at 2 h after inoculation (Figure 7). Heat stress and heat stress followed by Bgh inoculation suppressed the expression of *HvBI-1* at 0, 2, and 6 h after inoculation in both susceptible and resistant lines, as compared to plants held at an optimal temperature (20 °C) (Figure 7). 

Overall, in Bgh-resistant barley plants held at an optimal temperature (20 °C), the expression of the investigated stress/defense genes was activated at early time points after Bgh inoculation, while in the susceptible line, the expression of these genes either did not change or was suppressed during this initial stage (2 and 6 h) of pathogen infection. However, heat stress suppressed and/or delayed the expression of all mentioned genes, and this delay in defense gene expression may contribute to an enhanced susceptibility to Bgh (i.e., an increase in Bgh symptoms and biomass) in all investigated lines.

## 3. Discussion

Heat stress may have a profound influence on plant–pathogen interactions [1]. Our results showed that pre-exposure to 35 °C heat stress for different durations significantly increase powdery mildew (*Blumeria graminis* f. sp. *hordei* A6, Bgh) symptoms and biomass in resistant near-isogenic lines of barley (*Hordeum vulgare*) cv. Ingrid containing different resistance genes (*Mla12*, *Mlg*, *mlo5*). Furthermore, heat stress enhanced susceptibility in the susceptible line (WT). Heat stress of shorter durations (from 1 min in Mla12, 1 h in mlo5, and 2 h in Mlg lines) successfully increased Bgh biomass in resistant lines, however, in the susceptible line (WT), only the effect of prolonged (48 h and 120 h) heat stress increased the intensity of symptoms and biomass. In line with our experiments, others have demonstrated that a short-term pre-exposure to a high temperature (heat shock, e.g., submerging plants in 49 °C water for 20 s) may also confer enhanced susceptibility of these Bgh-resistant near-isogenic barley lines to Bgh [22]. In contrast, in susceptible barley, a heat shock (30–40 s at 50 °C) followed by immediate Bgh inoculation significantly reduced powdery mildew infection [24,25]. However, these results cannot be directly compared with the results obtained by us, as both the applied temperature and the method of heat treatment differed. A heat shock (55 °C for 45 s, 30 min before inoculation) transiently inhibited Mla-mediated HR to Bgh in barley coleoptiles [30]. We experienced something similar in Mla12 barley cv. Ingrid, where heat stress at 35 °C reduced local necrotic symptoms (HR) and, simultaneously, powdery mildew symptoms appeared in first leaves inoculated with Bgh. Schwarzbach [26] reported that exposure to a high temperature (36 °C for 30 min, 60 min, and 120 min durations) confers an enhanced susceptibility to Bgh in both genetically resistant (mlo) and susceptible barley cultivars [26]. Although our results confirmed that exposures to one and two hours of heat stress at 35 °C breaks mlo-mediated resistance, we have also shown that only a prolonged (48 and 120 h) heat stress may enhance susceptibility in the genetically susceptible Mlo line. Importantly, in our assays, we used intact barley plants as opposed to the experiments of Schwarzbach [26], where leaf segments were placed on agar medium, a likely cause of differential reactions of heat-stressed barley (35–36 °C for 1 and 2 h) to Bgh in the susceptible line. Recently, we found that prolonged heat stress pre-exposure (35 °C for 24, 48, and 120 h) confers enhanced susceptibility to Bgh in a susceptible barley line, while a resistant line retains its pathogen resistance [28]. Defense gene expression patterns following heat stress and Bgh inoculation were markedly different in the resistant and susceptible lines. In contrast, in the present investigation, heat stress converted powdery mildew resistance to susceptibility in Mla12, Mlg, and mlo5 lines and further enhanced susceptibility in WT plants. Accordingly, the expression of defense genes displayed a similar trend in the resistant and susceptible lines following heat stress and Bgh inoculation.

Interestingly, we found that the amount of fungal biomass in Mla12 barley is significantly higher even at 20 °C (without heat stress) than in the other two resistant lines (Mlg and mlo5). Higher Bgh biomass in Mla12 barley is presumably related to the slowness of HR as compared to other forms of resistance mediated by *Mlg* and *mlo5* resistance genes. It is known that Bgh penetrates the epidermal cells of Mla12 and susceptible Mlo barley, while effective papillae formation in Mlg and mlo5 lines prevent fungal penetration during the first 16 h of infection [20]. However, in Mla12 lines, Bgh can infect the surrounding cells and HR can only stop pathogen spread at ca. 36 h after infection [20]. This phenomenon could explain our observation that higher levels of Bgh biomass can be detected in the Mla12 line as compared to Mlg and mlo5 plants even without heat stress. Furthermore, this distinct difference between Mla12 and the other two resistant lines is also evident at high temperatures. 

Plant transcriptomes undergo extensive changes to cope with the influence of high temperatures, affecting not only physiology and growth but also the plant host’s ability to cope with pathogens [31,32]. Therefore, we investigated the expression of various plant defense genes in Bgh-infected resistant and susceptible barley plants pre-exposed to heat stress. 

Heat shock proteins (HSPs) are ubiquitous proteins found in plant and animal cells that accumulate rapidly under rising temperatures to minimize damage caused by heat [32]. Heat shock proteins were originally described in connection with heat stress [33,34]; however, they play an equally important role in pathogen-initiated plant defense signaling and disease resistance [35]. It has been described that Bgh inoculation induces the expression of the *Hsp90* homologue *HvGRP94* at early time points in barley cv. Pallas containing the *Mla* resistance gene [36]. Similarly, in our study, the expression of *HvHsp90-1* was induced transiently at two hours after inoculation in cv. Ingrid Mla12. However, we have also shown that the transient induction of *HvHSP90-1* is induced not only in the line containing the *Mla12* resistance (R) gene but also in Ingrid barley containing *Mlg* and *mlo5* R genes but not in the susceptible (WT) line. Others demonstrated that silencing of *Hsp90* breaks the *Mla13*-mediated resistance to Bgh in barley, indicating a functional role of *Hsp90* in resistance to Bgh [37]. Our results showed that 48 h of heat stress at 35 °C induces the expression of *HvHsp90-1* in barley, however, the expression drops drastically within 2 h after heat stress regardless of the presence or absence of powdery mildew. The lack of the transient induction of *HvHsp90-1* following heat stress is associated with the appearance of Bgh symptoms in resistant plants. The initial step of the defense signaling pathway is the recognition of pathogens by different plant receptors. Most of the plant resistance genes encode nucleotide-binding domain and leucine-rich repeat-containing (NLR) proteins that function as immune receptors. It has been found that Required for Mla12 resistance 1, Suppressor of the g2 allele of skp1, and Hsp-90 are indispensable for normal pathogen recognition due to the stabilization of NLR proteins such as the product of the *Mla12* resistance gene [38]. In our experiments, the early transient induction of *HvHsp90-1* observed in different barley cv. Ingrid lines resistant to BghA6 may play a similar role by activating the NLR receptor(s) recognizing Bgh. 

Heat stress often leads to the production of reactive oxygen species (ROS) in plant tissues [39,40]. Furthermore, ROS play an important signaling role during pathogen infection [41,42,43]. Therefore, controlling ROS during simultaneous pathogen infection and heat stress could be critical for successful plant survival. Respiratory burst oxidase homologue (RBOH) genes encode plasma membrane-bound proteins with NADPH-oxidase activity, and both pathogen recognition [44] and the sensing of high-temperature stress [39] is associated with RBOH-dependent ROS generation. In the present study, we have shown that in Bgh-infected plants held at a normal temperature (20 °C), an early induction of the barley *HvRBOHF2* gene is characteristic of the Bgh-resistant lines (Mla12, Mlg, and mlo5) but not the susceptible (WT) line. Furthermore, we have demonstrated that a prolonged heat stress with or without Bgh infection suppresses and delays the expression of the barley *HvRBOHF2* gene in both Bgh-resistant and -susceptible barley lines, as compared to Bgh-infected plants held at 20 °C. In line with our results, heat stress suppressed the expression of *TaRBOHF* in wheat [45]. Furthermore, transgenic barley with a silenced *HvRBOHF2* gene showed an enhanced susceptibility to the penetration of Bgh [46,47], demonstrating the role of HvRBOHF2 in the resistance of barley to Bgh. In fact, in tobacco kept at higher temperatures (30 °C) a reduced ROS generation and down-regulation of *NtRBOHD*, a functional homolog of *HvRBOHF2,* is correlated with the suppression of resistance to *Tobacco mosaic virus* [48]. This suggests that a reduced or delayed expression of *HvRBOHF2* during prolonged heat stress in both resistant and susceptible barley lines contributes to susceptibility to Bgh.

Programmed cell death (PCD) is defined as a sequence of genetically controlled developmental and stress-responsive events that direct the plant cell to die [49]. Pathogen infection induces PCD [50,51,52] as well as unfavorable environmental conditions such as heat stress [40,53]. The BAX Inhibitor-1 (BI-1) protein is a universal PCD inhibitor in eukaryotes including plants [54,55]. Our results showed that the expression of *HvBI-1* in response to Bgh is only slightly induced in the susceptible line (WT) held at 20 °C. On the other hand, in the resistant lines, an early, significant induction of *HvBI-1* was characteristic starting at 2 h after inoculation. Hückelhoven et al. [56] obtained similar results since an early enhancement of *HvBI-1* expression following Bgh infection was evident in resistant barley cultivars, especially in those genotypes (*Mla12*, *Mlg*) that undergo a hypersensitive resistance (HR) associated with PCD [56]. Furthermore, barley *BI-1* functions as a Bgh susceptibility factor, likely due to the suppression of plant PCD, since Bgh is an obligate biotrophic pathogen preferring live host tissues [57,58,59]. Here we demonstrate that heat stress suppresses and delays early *HvBI-1* expression in both resistant and susceptible barley lines regardless whether or not the plants are infected with Bgh, implying that BI-1 may contribute to plant tolerance to heat stress. In fact, Arabidopsis *BI* mutants display an enhanced sensitivity to heat-shock-induced PCD [60], and overexpressing the *CaBI-1* gene of pepper (*Capsicum annuum*) in tobacco (*Nicotiana tabacum*) significantly improved tolerance to abiotic stresses such as high temperature, salinity, and drought [61]. It is plausible that heat stress induces PCD in both the resistant and susceptible barley lines used in our study. Interestingly, we did not observe the appearance of necrotic symptoms characteristic of PCD on heat-treated plants. However, *HvBI-1* is activated in all lines between 24 and 48 h after heat stress regardless of Bgh infection; therefore, *HvBI-1* induction may contribute to the absence of necrotic symptoms. Nevertheless, our results suggest that the primary role of *HvBI-1* expression in both Bgh-resistant and -susceptible barley lines is the suppression of PCD, rather than controlling pathogen resistance. However, in certain plant–pathogen interactions BI-1 may play a role not only in PCD-inhibition but also in conferring disease resistance [62]. For example, the silencing of wheat *BI-1* turned an HR-type resistance into partial susceptibility during infection by the rust pathogen *Puccinia striiformis* f. sp. *tritici* [63].

The activation of pathogenesis-related (*PR*) genes and the accumulation of *PR*-encoded proteins in pathogen-attacked plants are well-known [64]. Based on their biochemical and biological functions, PR proteins are classified into 17 families [65]. Enhanced expression of several *PR* genes, e.g., *PR1,* is induced in infected wheat plants resistant to powdery mildew but not in susceptible cultivars [66]. Furthermore, plant PR1 proteins exhibit sterol binding activity, and inhibiting fungal sterols can also be potentially effective against pathogenic fungi such as powdery mildew [64]. The barley PR1-b protein has been shown to contribute to penetration resistance to Bgh, since transient silencing of *PR1-b* enhanced the penetration efficiency of powdery mildew in attacked epidermal cells [67]. In addition, another PR protein, PR17c, is also required for penetration resistance of barley to Bgh [68]. CSEP0055, a Bgh effector candidate, interacts with both PR1-b and PR17c, thus contributing to enhanced virulence and implying a possible suppression of these PR proteins by Bgh [68]. In the present study, we have shown that a prolonged heat stress drastically reduced and delayed the expression of *HvPR1-b* in both resistant and susceptible barley lines. The heat-stress-induced suppression of *HvPR1-b* may contribute to the appearance of Bgh symptoms in resistant lines and the enhanced Bgh symptoms in susceptible WT plants. 

Overall, high-temperature stress enhanced powdery mildew symptoms and biomass in both susceptible (WT) and resistant (Mla12, Mlg, and mlo5) cv. Ingrid barley near-isogenic lines. A possible reason for this increased pathogen susceptibility is the heat-induced suppression and delay in barley defense gene expression. Our findings may have profound implications in the face of global climate change tendencies, implying that breeding crops for tolerance to combined stresses (i.e., abiotic and biotic) should certainly be a primary task in the near future. 

## 4. Materials and Methods

### 4.1. Plant Materials and Powdery Mildew Infection

Barley (*Hordeum vulgare* L.) cultivar Ingrid and its near-isogenic lines containing different powdery mildew resistance genes (*mlo5; Mla12*; *Mlg*) were used in our experiments. Mla12, mlo5, and Mlg lines are resistant to barley powdery mildew *Blumeria graminis* (DC) Speer f. sp. *hordei* Em. Marchal, race A6 (Bgh) while the wildtype (WT) Mlo line is susceptible. Plants grown from ca. 30 seeds sown into two pots per treatment were kept in versatile environmental chambers (20 °C, 60% relative humidity, 16 h light/8 h dark photoperiod with a light intensity of 100 μmol m^−2^ s^−1^). For Bgh inoculation, conidia from heavily infected barley were dusted equally onto the primary leaves of 13 day-old barley seedlings of the genotypes listed above [7]. Inoculated plants displayed an inoculation density of about 50 conidia mm^−2^. Bgh was maintained on susceptible barley host plants (cv. Ingrid *Mlo*) in versatile environmental chambers under the conditions described above. 

### 4.2. Heat Stress

Barley plants were artificially exposed to a temperature of 35 °C (heat stress) at 80–85% relative humidity with daily watering prior to inoculation with Bgh in versatile environmental chambers with a 16 h light/8 h dark photoperiod and a light intensity of 100 μmol m^−2^ s^−1^. As a control, plants were kept at a constant 20 °C as described previously [22,26]. The reason for selecting 35 °C is that this temperature occurs rather often under field conditions in Central Europe, typically in growth periods when barley is exposed to Bgh (i.e., in May and June). The duration of heat stress varied between 30 s and 5 days (30 s, 1 min, 1 h, 2 h, 6 h, 24 h, 48 h, 120 h). During the 8 h dark period, the temperature was always decreased to 25 °C in order to better simulate field conditions. Powdery mildew inoculation of heat-stressed plants was performed as described above, immediately following heat treatment. 

### 4.3. Evaluation of Powdery Mildew Symptoms Caused by Bgh

The formation of powdery mildew symptoms on 10 randomly selected primary leaves per treatment of Bgh-inoculated plants was visually evaluated 7 days after inoculation (DAI). Disease severity was estimated as the percentage of area with powdery mildew symptoms per leaf. Inoculated primary leaves for each plant were evaluated. Three independent biological experiments were conducted, and 360 plants per experiment were assessed in the following arrangement: four barley lines (wild type, Mla12, mlo5, and Mlg) and nine different heat treatments (20 °C and 35 °C for 30 sec, 1 min, 1 h, 2 h, 6 h, 24 h, 48 h, and 120 h), with ten plants/treatment/barley line. 

### 4.4. Quantitative Analyses of Powdery Mildew Biomass 

For the quantification of Bgh biomass, barley leaf tissue samples (5 primary leaves from 5 individual plants randomly selected and pooled per treatment) were sampled from plants at 7 DAI, following symptomatic evaluation, in liquid nitrogen. To analyze Bgh biomass, a protocol of reverse transcription followed by quantitative real-time polymerase chain reaction (RT-qPCR) was used as described previously for barley-Bgh and wheat-*Puccinia striiformis* f. sp. *tritici* interactions [28,69]. Collected leaf tissue was ground in liquid nitrogen. Total RNA (including plant and Bgh RNAs) was isolated by the Plant Total RNA Extraction Miniprep System Kit according to the manufacturer’s instructions (Viogene, Sunnyvale, CA, USA). Following RNA isolation, samples were subjected to DNAse I treatment with RQ1 RNase-Free DNase (Promega, Madison, WI, USA). Analysis of RNA quantity and quality (260/280 and 260/230 ratios) was conducted by a MaestroNano Spectrophotometer (Maestrogen, Hsinchu City, Taiwan) and total RNA degradation was also checked by formaldehyde agarose gel electrophoresis. One microgram of total RNA per sample was used for reverse transcription (RT). RT was carried out with a RevertAid™ H^−^ cDNA Synthesis Kit (Thermo Fisher Scientific Baltics UAB, Vilnius, Lithuania) according to the instructions of the manufacturer. As a negative control, a pool of randomly selected RNA samples was applied to which no reverse transcriptase was added. Assaying the relative expression of *Glyceraldehyde 3-phosphate dehydrogenase* (*BgGAPDH*) as an optimal Bgh gene for RT-qPCR during the infection cycle [70], along with the barley reference gene *Ubiquitin* (*HvUbi*), was conducted with the 2X SYBR FAST Readymix reagent (KAPA Biosystems, Wilmington, MA, USA). The qPCR reactions were carried out as described by Höller et al. [71]. In brief, the PCR reaction mix contained 7.5 μL KAPA SYBR FAST qPCR Master Mix (2X), 0.75 μL of 5 μM forward and reverse primers each, 3.5 μLof PCR-grade water, and 2.5 μL of 20-fold diluted cDNA in a total reaction volume of 15 μL. DNA amplifications were performed in a Bio-Rad CFX-96 real-time thermocycler (Bio-Rad, Hercules, CA, USA), running a standard program (95 °C for 2 min, 40 cycles at 95 °C for 10 s, 60 °C for 10 s, and 72 °C for 10 s) followed by a melting curve analysis to determine amplicon specificity using a temperature range of 65 to 95 °C with increments of 0.5 °C. Gene expression was normalized to a barley *Ubiquitin* gene (*HvUbi*) as a reference. Previous research has demonstrated that *HvUbi* is a reliable reference gene for detecting gene expression changes in barley exposed to either powdery mildew infection, heat, or drought stress [7,46,70,72,73]. Testing the suitability of *HvUbi* as a reference gene was performed by analysis of cycle threshold (CT) variation in response to heat treatments and Bgh infection. Significant changes were not observed in CT values (mean ± standard deviation, SD) for *HvUbi* during treatments. All reactions were performed using three independent biological experiments with three technical replicates per biological sample. In each run, water-only controls and non-reverse-transcribed RNA were used as negative controls. The primer efficiencies for the genes tested were between 101–104%. Changes in gene expression were calculated using the 2^−ΔΔCT^ method [74]. For primers used in qPCR, see Table 1. Oligonucleotide primers specific to *HvUbi* were designed with the aid of the Primer Premier 5 program (PREMIER Biosoft International, San Francisco, CA, USA), while primers for *BgGAPDH* have been described previously [70].

### 4.5. Quantitative Analyses of Plant Defense Gene Expression

Samples for assaying barley defense gene expression (5 primary leaves from 5 individual plants randomly selected and pooled per treatment) were taken at five time points after heat stress immediately followed by Bgh inoculation (i.e., 0, 2, 6, 24, and 48 h after Bgh inoculation) and stored at −70 °C. To analyze the defense gene expression of barley. a protocol of reverse transcription followed by quantitative real-time polymerase chain reaction (RT-qPCR) was used. Methods used during the analysis (RNA isolation, RT, and qPCR) are described in detail in the previous section. During qPCR assay, the defense gene expression of barley was normalized to a barley *Ubiquitin* gene (*HvUbi*) as a reference. Changes in gene expression were calculated using the 2^−ΔΔCT^ method [74]. For primers used in qPCR, see Table 1. Oligonucleotide primers (*HvUbi*, *HvBI-1*, *HvPR1-b,* and *HvRBOHF2*) were designed with the aid of the Primer Premier 5 program (PREMIER Biosoft International, San Francisco, CA, USA) except for *HvHSP90-1* [75].

**Table 1:** Oligonucleotide primers used in qPCR.

Accession Number	Gene	Sequence 5′-3′		Amplicon Length	Primer Efficiency
**CAUH01004767**	*Glyceraldehyde* *3-phosphate* *dehydrogenase* (*BgGAPDH*)	Fw	GGAGCCGAGTACATAGTAGAGT	105 bp	104%
		Rev	GGAGGGTGCCGAAATGATAAC		
M60175	*Ubiquitin* (*HvUbi*)	Fw	ACCCTCGCCGACTACAACAT	240 bp	102%
		Rev	CAGTAGTGGCGGTCGAAGTG		
XM045113197	*Heat shock protein 90-1* *(HvHsp90-1)*	Fw	ACAAGAACGACAAGTCCGTCAA	119 bp	104%
		Rev	GAGCATGCGGTGGATCCT		
AJ290421	*BAX inhibitor-1* (*HvBI-1*)	Fw	ATGTTCTCGGTGCCAGTCT	409 bp	101%
		Rev	GGGCGTGCTTGATGTAGTC		
X74940	*Pathogenesis related -1b* (*HvPR1-b*)	Fw	GGACTACGACTACGGCTCCA	150 bp	104%
		Rev	GGCTCGTAGTTGCAGGTGAT		
EU566856.1	*Respiratory burst oxidase homologue F2* (*HvRBOHF2*)	Fw	TGCTCGGTCAGCACTC	175 bp	104%
		Rev	TCCGCAATAGAACACTCC		

Abbreviations: Bg, *Blumeria graminis* f. sp. *hordei*; Hv, *Hordeum vulgare*; Fw, forward primer; Rev, reverse primer.

### 4.6. Statistical Analyses

Statistical analyses were conducted using the Statistica 13 software (TIBCO Software, Palo Alto, CA, USA). Powdery mildew coverage of leaves and relative gene expression values were log-transformed to achieve homogeneity of variances (assessed by Bartlett’s test). Analysis of variance (ANOVA) and Tukey’s post-hoc test were employed, and differences at *p* < 0.05 were considered statistically significant.

## Data Availability

Not applicable.
